# Image Resolution in the Digital Era: Notion and Clinical Implications

**Published:** 2014-12

**Authors:** Vahid Rakhshan

**Affiliations:** Iranian Tissue Bank and Research Center, Tehran University of Medical Sciences, Dept. of Dental Anatomy and Morphology, Dental Branch, Islamic Azad University, Tehran, Iran.

**Keywords:** Dental Imaging, Computerized Imaging, DPI Resolution, Spatial Resolution, Calibration

## Abstract

Digital radiographs need additional metadata in order to be accurate when being converted to analog media. Resolution is a major reason of failures in proper printing or digitizing the images. This letter shortly explains the overlooked pitfalls of digital radiography and photography in dental practice, and briefly instructs the reader how to avoid or rectify common problems associated with resolution calibration of digital radiographs.

## Dear Editor

Digital imaging has been extremely beneficial to dental practice. Compared to conventional film-based radiographs, their digital alternatives need less X-ray exposure and preparation time, [1] and are much more convenient to handle, store, copy, and share by dentists, radiologists, and patients.[1-2] Moreover, digital radiographs and photographs might provide better accuracy and reproducibility, as dentists can use features of image editor programs (such as digital zoom and color/brightness editing tools) to make the image reading more comfortable ,though with a similar or even better accuracy. [1-2] Hence, practice and research in dentistry are relying progressively on the new format. This, however, can introduce some difficulties to these fields regarding the machine-human interfaces. This is because images may need to be digitized, edited, and printed, all of which can produce some artifacts that should be avoided.

Spatial resolution is best described by the modulation transfer function (MTF), since this clearly relates perceptible contrast to spatial frequency in an image. MTF is calculated from the line-spread function (LSF), and is the method of characterizing the spatial response of an imaging system. [3-5] It describes how well an imaging system performs in depiction of fine structures with minimal blur. [6] In digital radiography, the MTF at either 50% or 10% modulation is the internationally accepted measure for spatial resolution since they produce the highest resolutions. [7] Line pairs per mm might also be acceptable. Actually, neither pixel size nor DPI tells much about spatial resolution, as the latter refers to the frequencies in an image that can be perceived and was measured in line pairs/mm in earlier days. Nevertheless, the notion of DPI resolution acts like Achilles’ heel when it comes to digital radiography in dentistry.

DPI resolution is challenging for many dental practitioners, and even some academic radiologists. Therefore, in the computer era, a simple introduction on this matter to dentists is lacking and thus can be helpful. The following letter tries to cover this issue as well as some other items which were overlooked in the field of dental imaging. 

## Pixel resolution versus DPI resolution

A computer program only works when properly instructed by the user. DPI is an analog reflection of the way an image is broken down for printing physically or displayed virtually. Pixel or voxel resolution is determined by various elements in the imaging chain, including but not restricted to the detector elements, plus the line spread of data due to some issues such as scintillator usage and detector configuration. Pixel resolution (or pixel size) denotes the number of pixels in an image (referring to its image size) while spatial resolution identifies the graphical quality of the image. A megapixel is a notion currently overused by companies to advertise the resolution and incorrectly the quality of digital images taken with their products. Although megapixel points to some sort of resolution (like the pixel resolution), this kind of resolution actually means the size of an image and not necessarily its quality. The names “pixel size”, “pixel dimension” or “pixel resolution” of a digital image equally refers to its size or the number of pixels in the picture, defined as X × Y, where X and Y represent the number of pixel-columns (image width) and pixel-rows (image height), respectively. The unit megapixel is simply calculated by dividing that size by one million. [8]

Interaction with machine-human interfaces (i.e., input/output devices such as scanners, cameras, monitor screen, or printers) introduces a form of spatial resolution called “DPI resolution”, or “pixel density”. It is measured by the units DPI (dots per inch) or PPI (pixels per inch) that depicts the number of dots on a square-inch span of the image. For instance, if the DPI of a cephalograph is 96, there will be 96×96 pixels in each square inch of the paper-printed cephalograph or on the digital cephalograph, at 100% zoom, seen on the screen, or while being measured using virtual rulers of image-editor programs. [8-10]

The point that makes the DPI important for dentists is that a fixed-size digital image with constant number of pixels can be scanned or printed, either on paper or virtually, at different sizes (for example, at the actual size or smaller). According to the following formula, the DPI value contributes to the actual/printed size of a digital radiograph (i.e., the size of that image in metric units in the real world [paper] or virtual world [simulated paper]): Printed size _(inch_^ 2 ^_)_ = Size (pixel resolution) _(pixel_^ 2 ^_)_ / DPI resolution _(DPI)_

**Figure 1:**
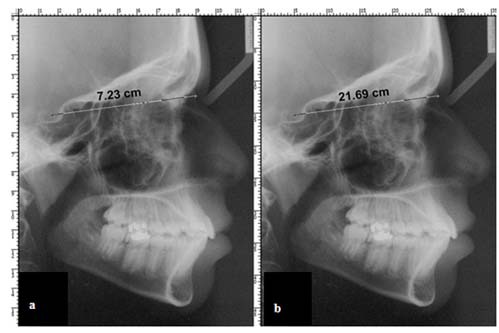
a: A cephalograph of 1416 × 1888 pixels2 with a resolution of 300 DPI. The to-be-printed size of this example radiograph (indicated by the Photoshop® rulers around the image) is 11.99 × 15.99 cm2. The SN measurement is reported 7.23 cm by the Photoshop® measuring tool. b: The same 1416 × 1888-pixel image, however, with a spatial resolution edited to 100 DPI. Its printing size is obviously different (35.97 × 47.96 cm2). The SN measurement is calculated as 21.69 cm using Photoshop®.

According to the above formula, when the pixel size and DPI value fit together, the pixels on the printed image would be exactly the same size as those in the digital image taken in the first place. The DPI value is firstly assigned to the file by the digitizer. For example, when a scanner device scans an image at a specific pixel size, it simply writes a single metadata value to the file as its DPI resolution value. Unlike the solid image size characteristic (width × height), this DPI resolution metadata may easily be changed or even removed by activities such as converting the image’s format (Bitmap format to JPEG), or editing it by some image-editor pogroms (saving a cropped portion of an image, enhancing sharpness and so on). In a constant-size radiograph, a greater DPI resolution leads to smaller pixels on the result of the output hardware (printer/monitor). [8-10] If there are two identical, same-size copies of a radiograph and we manipulate only their DPI metadata, even if both images’ sizes in pixels (pixel-size)  remain identical, the size of digital or paper-printed images in centimeter or inch will become different (Figure 1). Increasing the DPI value without consistently resizing the image will decrease the size of each printed dot in metric units, which would lead to a smaller printed image.

If one converts an image’s format (e.g. converting DICOM to JPEG), or edits it by image-editor pogroms (e.g. while saving cropped portion of an image), the DPI value may be lost (removed). When re-opening such a DPI-excluded image, or while checking its DPI resolution, a certain default value would be allocated to its DPI metadata by the opening image-editor program, or by the operating system. 

A point missing in the literature is that the value assigned to the DPI metadata by various programs is not standard and differs from case to case, which again can add to the confusion. This can be tested by opening a simple image in any format (like Bitmap), using MS Paint^®^ of Windows^®^ XP^®^, and saving it in another format (by the “Save As” option). An image without DPI value would be created. Checking its resolution in different ways would reveal different and contradicting DPI values for the same image, depending on the program that is used. For example, while Adobe^®^ Photoshop^®^ 8 and Gimpshop^®^ 2.2 would report “DPI=72” as its resolution, Paint.Net^®^ 3.3 and Microsoft^®^ Windows^®^ XP^®^ SP2 would inconsistently report “DPI=96” for that exactly same image file.

Modifying only the DPI value affects the image size in metric units, leading to unwanted changes in metric measurements, as my colleagues had experienced. Clinicians may need to know or rectify the DPI value in cases such as: 

Digitizing (like scanning or taking digital radiographs/photographs) or outputting images (like printing or illustrating on the monitor screen) with valid true-size metric measurements

Calculating or validating linear measurements on digital radiographs such as cephalometry linear measurements, gingival bone loss and so on.

Comparing measurements on different images of the same patient (on subtractive radiographs, pre- and post-treatment cephalographs and so on) which have been digitized with different DPI resolutions. 

The DPI resolution meta-value is modifiable through many image editor programs such as Adobe^®^ Photoshop^®^, Gimpshop^®^, Paint.Net^®^, or FastStone^®^ and so on.
